# A cationic surfactant-decorated liquid crystal-based sensor for sensitive detection of quinoline yellow

**DOI:** 10.1038/s41598-021-03788-6

**Published:** 2021-12-20

**Authors:** Fatemeh S. Mohseni-Shahri, Farid Moeinpour, Asma Verdian

**Affiliations:** 1 Department of Chemistry, Bandar Abbas Branch, Islamic Azad University, Bandar Abbas, Iran; 2 Department of Food Safety and Quality Control, Research Institute of Food Science and Technology (RIFST), Mashhad, Iran

**Keywords:** Chemistry, Analytical chemistry

## Abstract

Quinoline yellow (QY) is one of the popular synthetic food colorants and in food industry greatly used. Developing accurate and simple QY detection procedures is of major considerable importance in ensuring food safety. Hence, it is important to detect this food colorant effectively to reduce risk. Herein, an innovative liquid crystal (LC)-based sensor was designed for the label-free and ultra-sensitive detecting of the QY by means of a cationic surfactant-decorated LC interface. The nematic liquid crystal in touch with CTAB revealed a homeotropic alignment, when QY was injected into the LC-cell, the homeotropic alignment consequently altered to a planar one by electrostatic interactions between QY and CTAB. The designed LC-based sensor detected QY at the too much trace level as low as 0.5 fM with analogous selectivity. The suggested LC-based sensor is a rapid, convenient and simple procedure for label-free detection of QY in food industrial and safety control application.

## Introduction

Recently, there is a great enhancement in using synthetic dyes for many purposes like food additive, in cosmetics and drugs. These dyes are used to increase color, flavor and desirability in the food industries. Due to their lower sensitivity to pH, microbial contamination and less expense of production, they have an advantage over natural dyes^1^.

**Figure 1:**
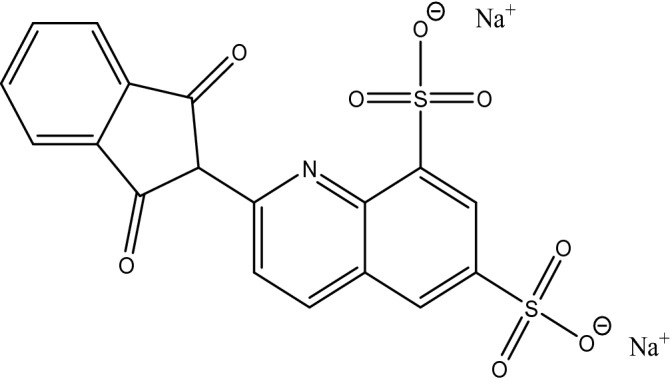
Chemical structure of quinoline yellow (QY).

Quinoline yellow (QY) (Fig. 1) is a synthetic yellow color dye that extensively used to give color to many food products, drugs, soft drinks, hair products and many cosmetic products. However, studies have shown that QY may cause in children skin allergies, irritation, respiratory, hyperactivity and skin rashes. There is possibility of hypersensitivity in persons who are allergenic to aspirin^2^. According to the premises mentioned, the European Food Safety Authority has diminished the passable intake of QY from 10 mg to 0.5 mg kg^−1^ of body weight^3^. So, it is entirely worthwhile and important to design a quick, sensitive and valid procedure for the detection of QY in food stuffs.

Several analytical methods have been developed for determining of QY, including spectrophotometric^4,5^, spectrofluorometric^6^, electrochemical methods^7–9^, high-performance liquid chromatography (HPLC)^10,11^ and UV–Vis detection^12^. However, these procedures are able to detect QY, but they have their own disadvantages. For example, HPLC is difficult, time-consuming and needed skilled operators, spectrophotometric method is not highly sensitive and affects by the interference of coexistent species. As well as hanging mercury drop electrode was used to QY determination, but the hanging mercury drop electrode is harmful to the environment.

In recent decade, liquid crystals (LCs) have attracted remarkable attention for the susceptible amplification and transduction of biomolecular phenomena into optical responses visible by the naked eye^13^. LC-based sensors have many significant benefits and do not need the use of labeled molecules and complex instrumentation^14^. LCs are interested materials with long-range directional order, optical anisotropy and fluidity. The alignment of LCs is extremely related to the alterations in the adjoining interface^15^. By LCs optical amplification, they can be turned into an incomparable optical probe for sensing reactions, such as peptide-lipid interactions, ligand-receptor binding and enzymatic reactions at the liquid crystal/aqueous interface. These interactions govern to a conversion from a homeotropic to planar orientation in the liquid crystal molecules. The liquid crystal molecules are orientated parallel and perpendicular to the surface in the planar and homeotropic alignments, respectively^16^. Previous researches have manifested that the respond of liquid crystal substances to exterior stimulation could be modulated by functionalizing them with surfactants, because self-assembling of the cationic surfactant at the liquid crystal-aqueous interface causes homeotropic anchoring (through sidelong hydrophobic interaction between hydrocarbon chains of cationic surfactant and LC)^17^.

**Figure 2:**
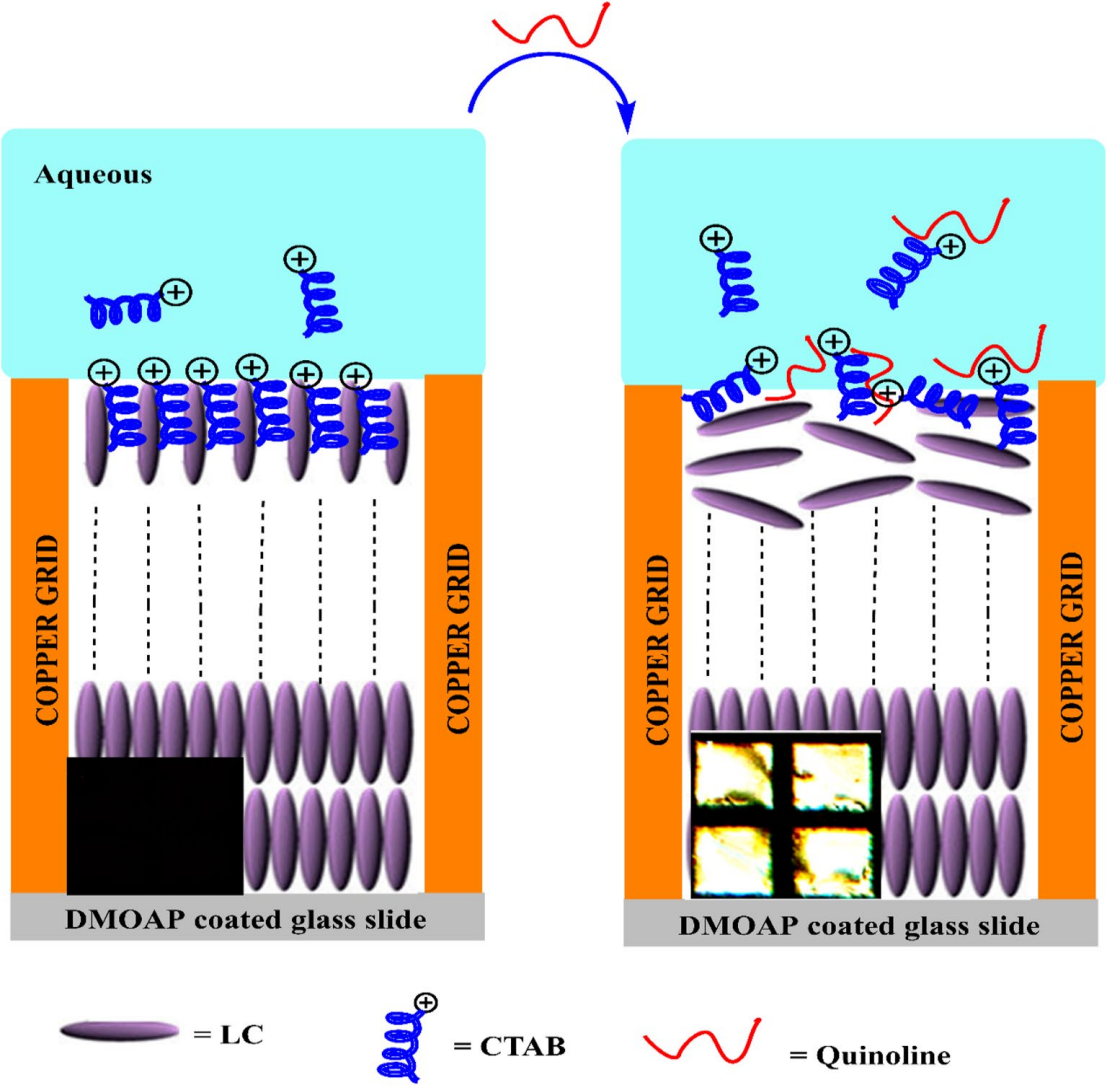
Schematic illustration of proposed plan for LC sensor for detection of QY based on self-assembly of CTAB at the interface.

In the present research, 5CB (4-cyano-4′-pentylbiphenyl, a thermotropic nematic liquid crystal)-filled TEM copper grids were used by a cationic surfactant coverage, cetyltrimethylammonium bromide (CTAB) at the liquid crystal/aqueous interface. We expected that aqueous solution of QY would interact with CTAB, would disarrange the original orientation of liquid crystal and cause a QY detection through the homeotropic to planar orientational alert of the 5CB (Fig. 2). To examine this idea, we first studied interaction of CTAB and QY by using absorption spectroscopy. Then we analyzed the limit of detection (LOD) and selectivity of this platform for QY detecting, based on the alteration in the optical pictures of liquid crystal.

## Materials and methods

### Materials

The standard glass microscope slides were bought from Fisher Scientific. Copper grids (150 mesh, 20 μm in thickness and 165 μm pitch), Nematic LC 4′-Pentyl-4-biphenylcarbonitrile (5CB) 98% were purchased from Sigma-Aldrich. Cetyltrimethylammonium bromide (CTAB) were obtained from Merck. Hydrogen peroxide 30% and Sulfuric acid 99.9% purchased from Sigma-Aldrich. Quinoline yellow was obtained from Dynemic Company (India). Deionized water has been used in the preparation of all aqueous solutions.

### UV–Vis spectroscopy

The UV–Vis spectra were registered at ambient temperature on a SPEKOL 1500 UV–Vis spectrophotometer supplied with 1 cm quartz cells. The slit width was adjusted to 5 nm and the wave-length range was 200–500 nm. QY was dissolved in the DI water and diluted to 5.0 μM. In measuring of each data point, 50 μl of the CTAB solution (4.0 mM) was added to 2 ml of the QY solution and then UV absorbance spectra were measured.

### Preparation of DMOAP-coated glass substrates

The glass slides were cleansed in piranha solution (30% H_2_O_2_ and 70% H_2_SO_4_) at 80 °C for 2 h and then the slides were washed thoroughly with HPLC grade water and ethanol, several times and dried under a flow of nitrogen and drying in thermostat oven at 110 °C for 3 h. The cleansed glass slides were then submersed in an aqueous solution comprising 0.35% (v/v) dimethyloctadecyl[3-(trimethoxysilyl) propyl] ammonium chloride (DMOAP) for 30 min and washed several times with HPLC grade water and ethanol. At the end, the DMOAP-covered glass was dried again under teeny stream of nitrogen and was retained in an oven at 110 °C for 1 h.

### Fabrication of LC-based sensor system

For the preparation of LC-sensor cell, a copper grid was put on a part of DMOAP-covered glass (5 mm × 5 mm) and loaded with 2 μL of 5CB which doped with CTAB (isotropic state at 40 °C). Then the excess of LCs removed with a clean capillary tube. Different concentrations of QY solutions were prepared and then, QY solutions were added into the liquid crystal-based sensor platform. The optical transmission of the 5CB was determined by a polarizing optical microscope (Motic, BA 400 B-POL, Spain) in transmission mode.

### Selectivity of LC sensor for QY detection

The liquid crystal-based sensor selectivity was checked with the aid of basic red 46, basic violet 16, basic yellow 28, navy blue, disperse yellow E-3G (dis E-3G) dyes (Concentration of each sample = 50 fM). Data are means ± SD, n = 3.

## Results and discussion

### Investigation on the interaction between QY and CTAB

**Figure 3:**
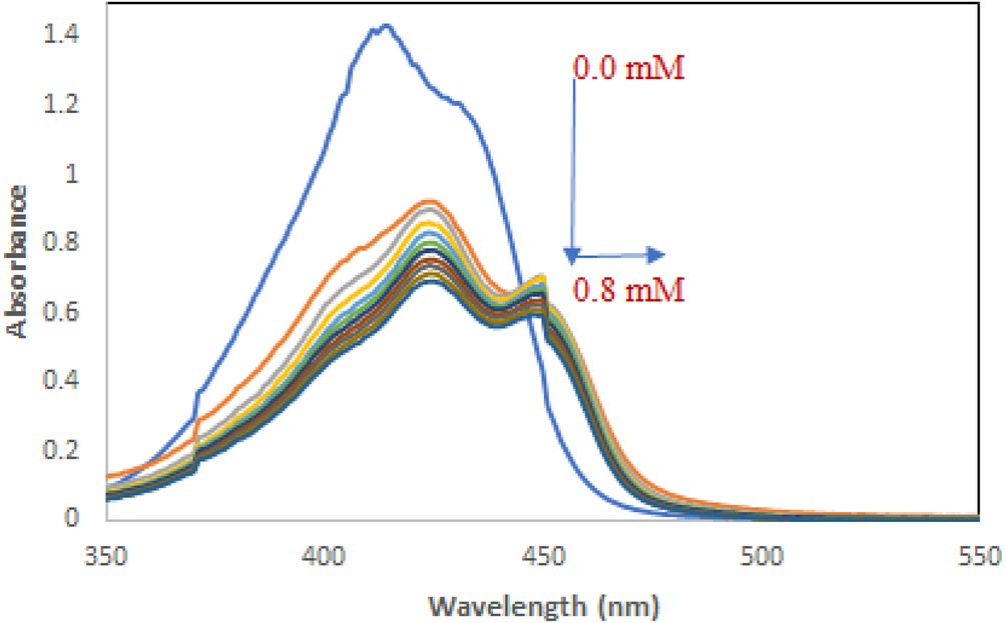
Ultraviolet–visible absorption spectra of QY in the presence of various concentrations of CTAB. [QY] = 5.0 μM, [CTAB] = 4.0 mM, T = 298 K.

To investigate whether CTAB could interact with QY or not, spectrophotometric data were employed. Figure 3 shows the UV–Vis absorption spectra of QY in the presence of various concentrations of CTAB. The absorbance band intensity of QY at 484 nm decreased with increasing concentration of CTAB and the peak position shows a moderate blue shift, suggesting the formation of QY-CTAB complex. This result guide us to deduce that CTAB could willingly interact with QY to yield related complex^18^.

### Optimization of CTAB concentration

**Figure 4:**
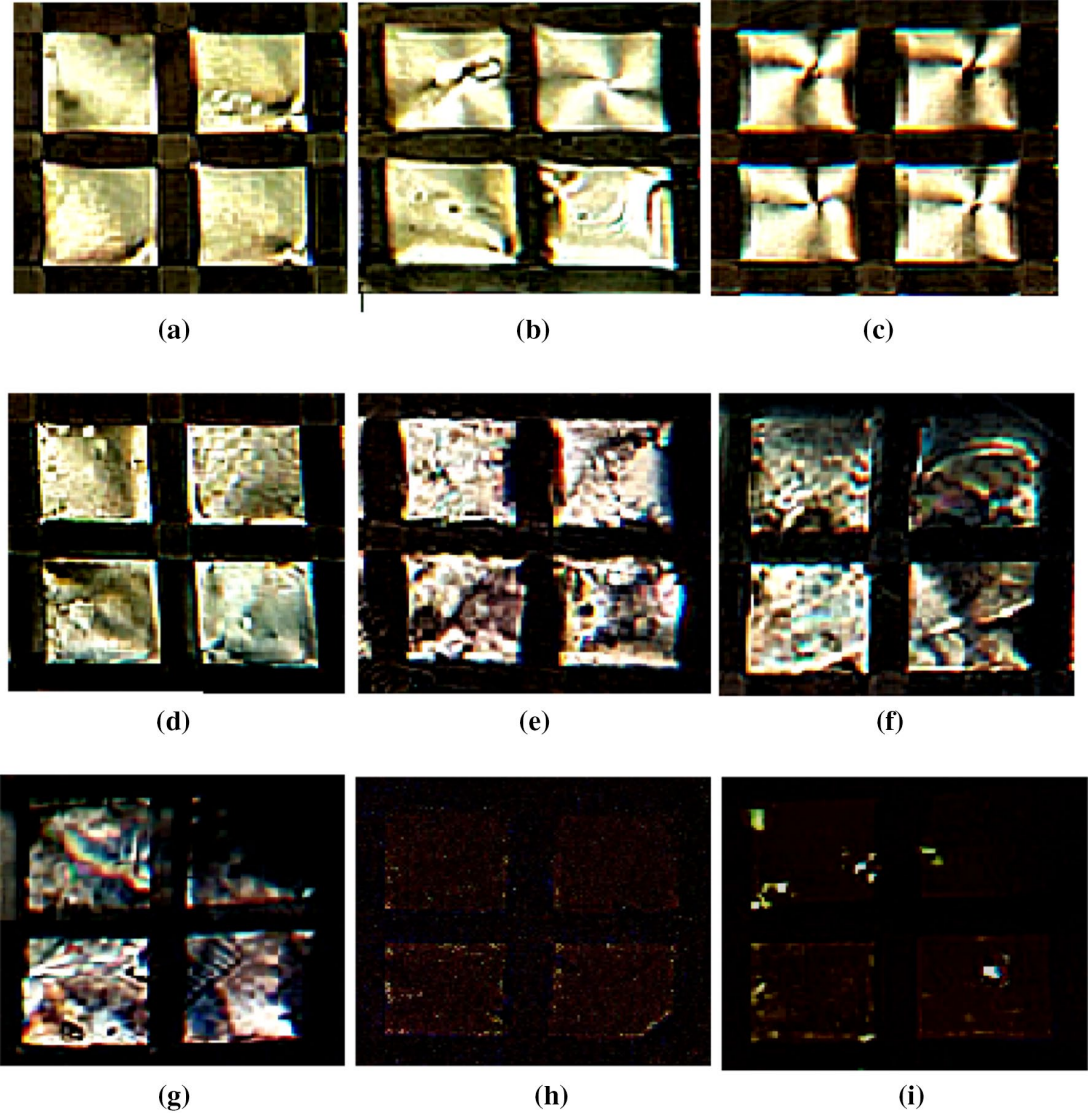
POM images (under crossed polarizers) of the LC cells at different concentration of CTAB = (**a**) 0, (**b**) 0.5, (**c**) 1.0, (**d**) 2.0, (**e**) 4.0 (**f**) 6.0, (**g**) 8.0, (**h)** 10.0 and (**i**) 12.0 mM, Scale bar: 100 µm.

Previous studies have shown that to regularize the alignment of the liquid crystals at the LC-aqueous interface, CTAB could be used^19,20^. Adding of CTAB onto the DMOAP-coated glass imparted the homeotropic regulation of liquid crystals via the hydrophobic interactions between the LCs and hydrocarbon chains of CTAB and causing dark optical appearances of the LCs. Hence, the minimum CTAB concentration adequate to operate the homeotropic anchoring of the liquid crystals should first be determined. For this reason, diverse concentrations of CTAB were injected to liquid crystal and incubated for 30 min. Then the polarized optical pictures of liquid crystals at various concentrations of CTAB were recorded. As indicated in Fig. 4, entirely dark in appearance of liquid crystals were seen when the CTAB concentration ≥ 10 mM, corresponding to the homeotropic anchoring of the liquid crystals at the interface. Hence, a CTAB concentration of 10 mM was considered as the optimum CTAB concentration in following experiments.

### Detection of QY by using LC-based sensor

When an aqueous QY solution was inserted at the LC/aqueous interface of liquid crystals, electrostatic interactions occurred between the anionic group of QY and cationic head groups of CTAB. The electrostatic pairing afforded the disruption of the self-assembly of CTAB at the interface and the primary homeotropic alignment of the liquid crystal molecules altered to a planar alignment. Following, we added QY solution into LC cell system to evaluate how the electrostatic interactions between CTAB and QY affect the orientations of LC and polarized optical microscope (POM) images^15^.

**Figure 5:**
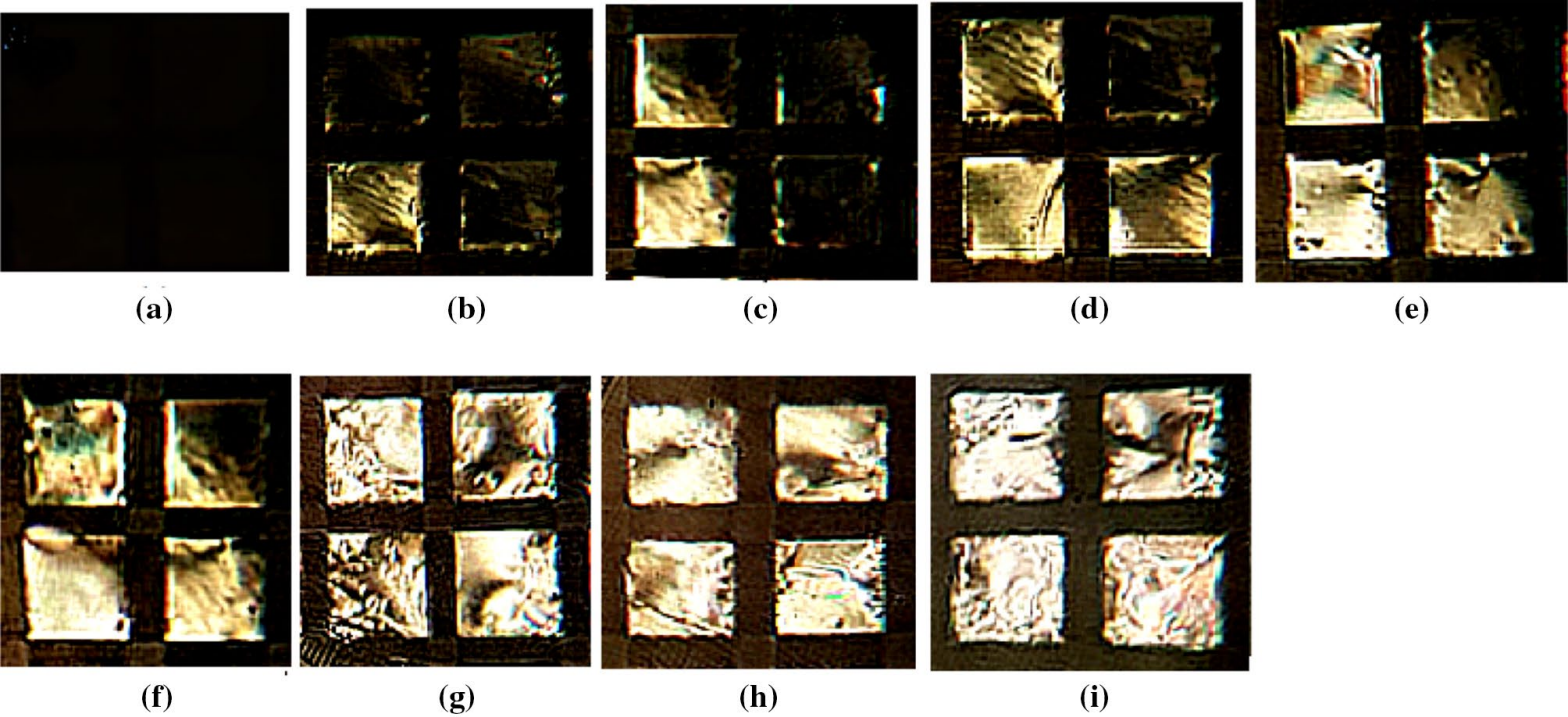
POM images of the CTAB-decorated aqueous/LC interfaces in contact with different concentrations of QY: (**a**) 0, (**b**) 0.05, (**c**) 0.5, (**d**) 5, (**e**) 50, (**f**) 500, (**g**) 5000, (**h**) 5 × 10^4^ and (**i**) 5 × 10^5^ fM, Scale bar: 100 µm.

Figure 5 indicates the polarized optical microscope pictures of the LC-cell in aqueous QY solutions at various QY concentrations. The bright regions in the images become greater with the concentrations of QY increasing from 0.05 to 5 × 10^5^ fM, confirms that the binding of QY to CTAB can change the initial homeotropic orientation of the LC-cell to a planar orientation.

**Figure 6:**
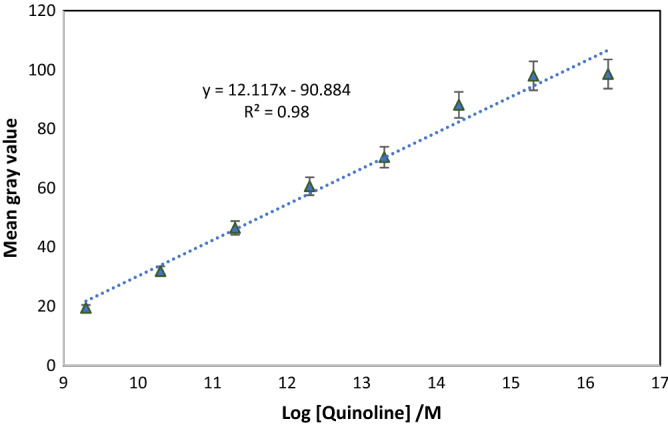
Graph representing mean gray value parameter as a function of QY concentration (n = 3).

To quantitatively elucidation of the developed sensor efficiency, the mean grey value parameter of the POMs was achieved by means of ImageJ software (NIH Freeware). Figure 6 demonstrates the correspondence between the mean grey value and the logarithm of QY concentration. As the results show the detection limit of QY in this study is about 0.5 fM. Extraordinarily, the LC-based sensor quantitatively detected QY with a too much low detection limit.

Compared to some available sensors for the QY detection, our assessment displays relative high sensitivity with a high performance as shown in Table 1.

**Table 1:** Comparison of the reported methods for the QY detection with presented LC sensor.

Detection method	LOD (μM)	Strategy	References
Electrochemical	1.05	Carbon nanotube-modified electrode	^21^
Electrochemical	0.027	Polyvinylpyrrolidone-modified electrode	^22^
Electrochemical	0.002	Graphene oxide modified grassy carbon electrode	^23^
Electrochemical	0.08	Polypyrrole/single-walled carbon nanotubes composites modified glass carbon electrode	^24^
Electrochemical	0.04	Manganese dioxide functionalized graphene	^25^
Electrochemical	0.004	Layer-by-layer fabricated multi-walled carbon nanotube	^26^
Electrochemical	0.0002	Hanging mercury drop electrode	^27^
Electrochemical	40.0	PVC-based graphite electrode	^9^
Liquid crystal	0.5 × 10^–9^	Homeotropic to tilted transition	This study

### Selectivity of LC-based sensor for QY detection

**Figure 7:**
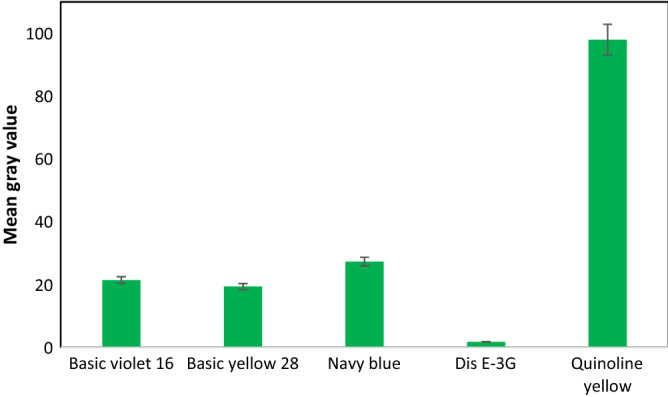
Selectivity of the LC-based sensor for the QY detection upon the addition of different common dyes (50 fM) under the optimal conditions.

The selectivity of the platform was assessed through basic red 46, basic violet 16, basic yellow 28, navy blue, dis E-3G dyes. The results in Fig. S1 shows that basic violet 16, basic yellow 28, navy blue and dis E-3G have no special binding to the QY sensor, therefore, the orientation of the liquid crystal molecules was conserved. Figure 7 shows the mean grey amounts of the various POMs. These outcomes confirm the selectivity of QY detection through designed liquid crystal-based sensor.

## Conclusion

We reported a new and simple LC sensor based on a cationic surfactant-decorated LC interface for the detecting of QY, a hazardous food colorant. QY could disrupt the organization of the CTAB monolayer at the liquid crystal interface, therewith causing alter of the LC responses from dark-to-bright appearance. The optimum concentration for CTAB to design the sensor was 10 mM. The developed sensor has high selectivity for QY with lowest detection limit 0.5 fM, based on the alter in orientation and optical properties of the LCs for QY detection. This study introduces a simple, label-free and low-cost sensor for the detection of QY which displays promising and potential perspectives in sensing approaches.

## Supplementary Information

Supplementary Figure S1.
