# Supplementary material for: A cationic surfactant-decorated liquid crystal-based sensor for sensitive detection of quinoline yellow
Source: Sci Rep. 2021 Dec 20;11:24264. doi: 10.1038/s41598-021-03788-6 (PMC8688477; doi:10.1038/s41598-021-03788-6)
Supplement: Supplementary file 1 — Supplementary Figure S1. [file 41598_2021_3788_MOESM1_ESM.docx]

**Supplementary information**


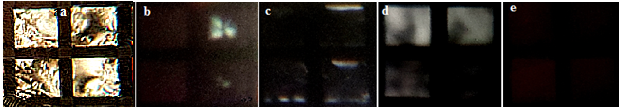


**Fig. S1** Selectivity test with the sensor immersed in the aqueous solution containing 50 fM of

(a) quinoline yellow, (b) basic violet 16, (c) basic yellow 28, (d) navy blue and (e) dis E-36,

Scale bar: 100 µm
